# Improving oxygen therapy for children and neonates in secondary hospitals in Nigeria: study protocol for a stepped-wedge cluster randomised trial

**DOI:** 10.1186/s13063-017-2241-8

**Published:** 2017-10-27

**Authors:** Hamish R. Graham, Adejumoke I. Ayede, Ayobami A. Bakare, Oladapo B. Oyewole, David Peel, Amy Gray, Barbara McPake, Eleanor Neal, Shamim Qazi, Rasa Izadnegahdar, Adegoke G. Falade, Trevor Duke

**Affiliations:** 1 Centre for International Child Health, University of Melbourne, MCRI, Royal Children’s Hospital, Level 2 East, 50 Flemington Road, Parkville, VIC 3052 Australia; 2 Department of Paediatrics, University College Hospital, Ibadan, Nigeria; 3 Department of Paediatrics, University of Ibadan, Ibadan, Nigeria; 4 Ashdown Consultants, Hartfield, England; 5 Nossal Institute for Global Health, University of Melbourne, Melbourne, VIC Australia; 6 Department of Maternal, Newborn, Child and Adolescent Health, World Health Organization, Geneva, Switzerland; 7 Bill and Melinda Gates Foundation, Seattle, WA USA

**Keywords:** Child, Infant, Oximetry, Solar energy, Quality improvement, Pneumonia, Stepped-wedge design, Theory-based evaluation, Developing countries

## Abstract

**Background:**

Oxygen is a life-saving, essential medicine that is important for the treatment of many common childhood conditions. Improved oxygen systems can reduce childhood pneumonia mortality substantially. However, providing oxygen to children is challenging, especially in small hospitals with weak infrastructure and low human resource capacity.

**Methods/design:**

This trial will evaluate the implementation of improved oxygen systems at secondary-level hospitals in southwest Nigeria. The improved oxygen system includes: a standardised equipment package; training of clinical and technical staff; infrastructure support (including improved power supply); and quality improvement activities such as supportive supervision. Phase 1 will involve the introduction of pulse oximetry alone; phase 2 will involve the introduction of the full, improved oxygen system package. We have based the intervention design on a theory-based analysis of previous oxygen projects, and used quality improvement principles, evidence-based teaching methods, and behaviour-change strategies.

We are using a stepped-wedge cluster randomised design with participating hospitals randomised to receive an improved oxygen system at 4-month steps (three hospitals per step). Our mixed-methods evaluation will evaluate effectiveness, impact, sustainability, process and fidelity. Our primary outcome measures are childhood pneumonia case fatality rate and inpatient neonatal mortality rate. Secondary outcome measures include a range of clinical, quality of care, technical, and health systems outcomes. The planned study duration is from 2015 to 2018.

**Discussion:**

Our study will provide quality evidence on the effectiveness of improved oxygen systems, and how to better implement and scale-up oxygen systems in resource-limited settings. Our results should have important implications for policy-makers, hospital administrators, and child health organisations in Africa and globally.

**Trial registration:**

Australian New Zealand Clinical Trials Registry: ACTRN12617000341325. Retrospectively registered on 6 March 2017.

**Electronic supplementary material:**

The online version of this article (doi:10.1186/s13063-017-2241-8) contains supplementary material, which is available to authorized users.

## Background

Oxygen therapy is an essential medication that is poorly available to sick children globally, despite clear evidence of benefit and wide clinical application [1]. As the primary treatment for hypoxaemia, oxygen therapy is relevant for a variety of clinical conditions including pneumonia and other lung conditions, sepsis, malaria, trauma, and neonatal conditions (including respiratory distress syndrome, asphyxia, and neonatal sepsis). Together, these conditions are responsible for 50% of child deaths globally [2], and hypoxaemia is both a common fatal complication and a significant independent risk factor for death. A recent systematic review estimated that hypoxaemia affects approximately 13% of children admitted to hospital with severe pneumonia, 20% of sick neonates and 10–15% of children with conditions such as malaria, meningitis, or convulsions [3].

Despite the enormous need for oxygen, its availability and use in hospitals globally is poor. Oxygen assessment from various countries shows that access to oxygen equipment, clinical guidelines, training, and technical support is lacking [4–8]. Improved oxygen systems can reduce hospital case fatality rates from childhood pneumonia by 35% [9], when implemented as part of a broad-based quality improvement package. However, experience from four decades of ‘oxygen projects’ shows that effective improvement of oxygen systems in low-resource settings is complex, requiring context-specific technical, clinical, and managerial solutions [10].

Data from various countries shows that improved oxygen use requires optimising both oxygen availability and the use of oxygen by healthcare workers. Achieving good oxygen access requires provision of quality, user-friendly equipment that functions in harsh conditions (e.g. high heat, humidity and dust) and systems to ensure equipment can be maintained, repaired, and replaced (including a system for sustained financing or cost-recovery) [10]. Projects in Egypt [11], the Gambia [12, 13] and Papua New Guinea [9, 14] provide successful examples of improving oxygen access through the use of appropriate equipment and supported by maintenance systems. Achieving good clinical use of oxygen requires skilled, motivated healthcare workers and a work environment that makes it easy to use oxygen well. Projects in Laos [15] and Papua New Guinea [9, 14] provide successful examples of improving healthcare worker use of oxygen through practical training, supportive supervision, and the implementation of evidence-based clinical guidelines. These and other oxygen projects demonstrate that improving oxygen therapy for children in hospitals requires addressing broader case management and care processes, and managerial and policy support [9, 14–17].

### Nigerian context

Nigeria is a populous middle-income country in sub-Saharan Africa that contributes to global child mortality disproportionately. Despite a decrease in child mortality since 1990 (U/5 mortality 213 to 117, 1990 to 2012), child, infant, and neonatal mortality remains high (117, 69 and 34 per 1000 live births, respectively) [18]. The biggest childhood killers are pneumonia (18%), malaria (14%), complications of prematurity (12%), birth asphyxia and trauma (11%), diarrhoeal diseases (10%), and neonatal sepsis (5%) [19].

Nigeria has a decentralised healthcare system with three levels of healthcare delivery: primary, secondary, and tertiary (plus alternative and traditional healthcare providers). Government health expenditure is minimal (7% of total government expenditure, among the lowest in the world) and 66% of total health expenditure is out-of-pocket expenditure, accounting for 9% of household expenditure on average [20]. Public secondary health facilities generally are managed by state government, which provide salary support; however, most day-to-day service activities are funded by patient fees [21]. Private and mission-based health services are common [21]. Nigeria has a large number of healthcare providers, comprising the largest human resource for health in Africa [22]. However, their distribution is weighted heavily towards large urban areas and there is a strong financial incentive to work at federally funded tertiary institutions over state-funded secondary health facilities [22]. Migration of healthcare workers out of Nigeria, and out of the Africa region, is a persistent challenge [22].

Limited data from southwest Nigeria suggests that hypoxaemia is common in children admitted to hospital [23], and access to oxygen therapy for children and newborns is a major challenge [24]. At one tertiary hospital, 29% of children under 5 years of age were hypoxaemic on admission, including 49% of pneumonia cases, 41% of sick neonates, and 14–30% of malaria cases [23, 25].

### Theoretical basis

We use a realist approach to project design, implementation, and evaluation. The realist approach seeks to understand *how* intervention(s) work to produce particular outcomes in a particular context [26]. As such, the realist approach is similar to other theory-based approaches in seeking to develop and test theories of how interventions work in particular contexts in order to inform future implementation in challenging real-world environments [27]. In planning this project, we reviewed experience from previous oxygen projects, including interviewing project coordinators about what they had done, why they chose that approach, and how it had worked (processes and outcomes) [10]. From this, we obtained an understanding of how various strategies have succeeded and failed in different contexts, and could make more informed decisions about how we would approach it in the Nigerian hospital context.

In designing the various components of our intervention, we drew heavily on theories of behaviour change, in particular Michie’s Theoretical Domains Framework (TDF) [28, 29] and the Behaviour Change Wheel (BCW) [30]. The TDF (and BCW) provides a framework for choosing particular behaviour-change strategies based on the behaviour being targeted and the barriers that need to be addressed. For the training activities, we used modern educational theories, particularly Merrill’s ‘first principles’ approach [31]. Merrill promotes a task-based, active learning approach that is structured around ‘demonstration’ (showing), ‘application’ (practice and feedback), ‘activation’ (builds on prior knowledge), and ‘integration’ (application in their particular context).

### Objectives

Given the clinical benefits of oxygen therapy, and the current poor access and use of oxygen therapy in Nigerian hospitals, we aim to implement an improved oxygen system in 12 Nigerian hospitals. We anticipate benefits in improved quality of care, and improved clinical outcomes for children (particularly children with pneumonia) and neonates. Our primary objective is to understand how to most effectively implement oxygen systems in complex, resource-limited environments. Our mixed-methods evaluation will evaluate effectiveness, impact, sustainability, process and fidelity.

## Methods/design

### Study design

**Fig. 1:**
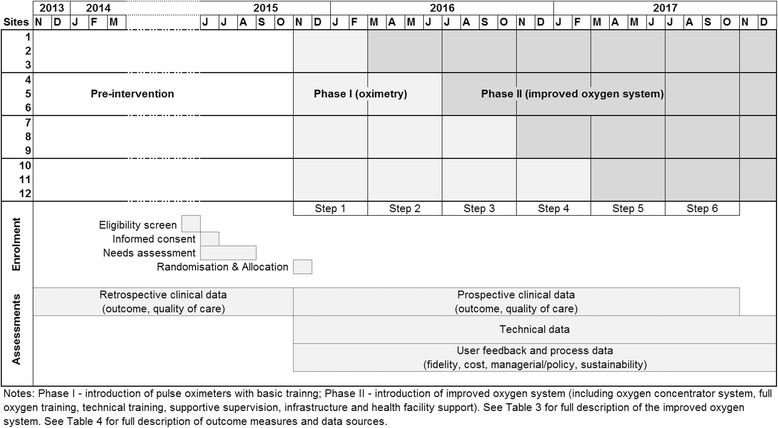
Standard Protocol Items: Recommendations for Interventional Trials (SPIRIT) Figure of stepped-wedge study design, illustrating stepped roll-out of the intervention, enrolment and data collection activities over time

This is a stepped-wedge cluster randomised field trial (cross-sectional). The study design and characteristics are summarised in Table 1 and Fig. 1 (see Additional file 1 for the Standard Protocol Items: Recommendations for Interventional Trials (SPIRIT) Checklist).

**Table 1:** Characteristics of the Nigeria Oxygen Implementation Project stepped-wedge cluster randomised trial

Trial characteristic	Definition
Cluster unit	Individual hospital. There are 12 clusters (hospitals)
Cluster randomisation	3 clusters (hospitals) randomised to receive the intervention at a particular time point. There are 4 cluster groups
Length of steps (observation periods)	4 months
Number of steps	6 steps
Description of steps	Step 1 – all hospitals are pre-intervention Steps 2, 3, 4 – intervention clusters are added sequentially Steps 5, 6 – all hospitals are intervention hospitals
Duration of trial	24 months
Pre-intervention period	4 to 16 months
Post-intervention period	8 to 20 months

We chose a stepped-wedge design as the most efficient and pragmatic trial design for our purposes, using methodology described by Hemming et al. [32–34]. Stepped-wedge designs can be useful for investigating the effects of an intervention that is likely to be beneficial in real-world environments. For this reason, it has been used increasingly for evaluating service delivery type interventions in developing countries, and is regarded as a pragmatic design suited to real-world settings [32]. We wanted all hospitals to receive the intervention and we had preliminary data suggesting that it was an effective intervention. We wanted to roll the intervention out sequentially, so it was not feasible to do parallel randomisation. We anticipated moderate intra-cluster correlation meaning that the stepped-wedge design would be relatively efficient. The pulse oximetry provision and training will be done before the full intervention as a ‘phase I’, as hypoxaemia data is necessary for evaluation of the full intervention.

We made a pragmatic decision to define clusters at the hospital level, as this was the natural level at which the intervention would be implemented and geographic separation between hospitals meant there was minimal risk of contamination. We considered clustering at the sub-hospital level (e.g. by ward), but rejected this due to the heterogeneity of ward arrangements, the large cross-over between wards, and the logistical complications that would have resulted. We randomised groups of three hospitals to receive the intervention at four different time-points (steps), spaced 4 months apart. This was based on practical considerations (i.e. how many hospitals could be practically set-up at a particular point in time), effect considerations (i.e. the effect should be realised within this time period), and statistical considerations (i.e. balancing the number of steps with the number of clusters within each step). We considered pairing clusters (hospitals), but rejected this due to the low number of included hospitals. We nominated a 3-week cross-over time for each hospital as it ‘stepped’ into the intervention, as this would be adequate time for the intervention to be fully implemented and pre-intervention patients to be discharged (to avoid contamination). We determined that data collection would be cross-sectional, measured at multiple points before and after the intervention at the level of individual patients (and anticipating a low rate of re-admission during the trial period).

### Study setting

This study is based in 12 secondary-level hospitals across four states (Oyo, Ondo, Ogun, and Osun) in southwest Nigeria (Table 2). In a three-tier health system, secondary-level hospitals have an important role in providing first-contact hospital care for the population. We wanted to conduct this study in hospitals that would be representative of secondary-level hospitals treating children in a challenging, high-mortality environment, to ensure that findings would be most relevant for future scale-up in Nigeria and sub-Saharan Africa more broadly.

**Table 2:** Characteristics of 12 secondary-level hospitals in southwest Nigeria: paediatric and neonatal wards (adapted from baseline needs assessment [8])

	1	2	3	4	5	6	7	8	9	10	11	12
Hospital type	State	Mission	Mission	State	State	State	State	State	Mission	Mission	Mission	State
Beds (child + neonatal)	13 (9 + 4)	32 (20 + 12)	63 (38 + 25)	36 (26 + 10)	46 (22 + 24)	60 (44 + 16)	48 (20 + 28)	36 (16 + 20)	20 (15 + 5)	14 (12 + 2)	70 (40 + 30)	25 (21 + 4)
Admissions (annual)	185	507	2343	238	791	849	3431	1945	431	344	2345	744
Child	180	444	1649	231	552	679	1959	1111	292	325	972	683
Neonate	5	63	693	7	239	170	1473	834	139	19	1373	61
Staffing												
Access to paediatrician	No^a^	No^a^	Yes^b^	No^a^	No	Yes	Yes	Yes	Yes	Yes^b^	Yes^b^	Yes
Medical (entire hospital)	7	4	6	7	12	17	16	11	5	6	4	2
Nursing (paediatric)	11	7	18	26	31	62	26	33 (3)	9 (2)	4	18	16 (2)
Power supply (h/day) power interruptions	6–12 hourly	6–12 hourly	12–18 hourly	6–12 hourly	6–12 hourly	12–18 hourly	12–18 hourly	6–12 hourly	>18 > daily	>18 > daily	12–18 hourly	6–12 hourly
Oxygen supply	Poor	Poor	Poor	Poor	Fair	Poor	Poor	Poor	Fair	Poor	Poor	Poor
Oxygen cylinders	Yes^d^	Yes^d^	Yes	Yes^d^	Yes	Yes	Yes^c^	Yes	Yes^d^	Yes^d^	Yes^d^	Yes^d^
Oxygen concentrators	No	No	Yes^e^	No	No	No	Yes^e^	Yes^e^	Yes	Yes^e^	Yes^e^	Yes^e^
Pulse oximeters	No	No	No	No	No	Yes	No	No	Yes	Yes	No	No
Maintenance capacity and procedures	Poor	Poor	Poor	Poor	Poor	Poor	Poor	Poor	Poor	Poor	Fair	Fair
Clinical guidelines, education and review	Poor	Fair	Poor	Poor	Fair	Poor	Good	Fair	Fair	Poor	Fair	Poor
Care systems												
Clinical records	Fair	Fair	Fair	Fair	Fair	Fair	Fair	Fair	Fair	Fair	Fair	Fair
Administrative records	Poor	Poor	Poor	Poor	Poor	Poor	Poor	Poor	Poor	Fair	Fair	Poor
Medication and supplies	Good	Good	Good	Good	Good	Good	Good	Good	Good	Good	Good	Good
Infection control	Good	Good	Good	Good	Good	Good	Good	Good	Good	Good	Good	Good
Neonatal resuscitation	Poor	Poor	Poor	Poor	Poor	Good	Good	Poor	Poor	Poor	Poor	Poor

Notes: ^a^Family medicine consultant, ^b^part-time, ^c^piped system connected to large oxygen cylinder, ^d^not present in paediatric areas, ^e^present but not fit for use. Summary indicators of oxygen supply and care systems include composite measures for oxygen supply reliability (presence of oxygen source, access to resupply, quantity to meet need), oxygen delivery devices (nasal prongs/catheter presence, access to resupply, quantity to meet need), clinical records (quality of case note and admission book documentation), administrative records (quality of medical record summary statistics procedures, documentation, and reporting), medication and supplies (checklist of essential items), infection control (availability and visible use of water and soap in clinical areas, reported infection control procedures), neonatal resuscitation (presence of resuscitation bag, neonatal mask, resuscitation table/cot)

In collaboration with the Oyo State Hospitals Management Board, we screened all government secondary health facilities in Oyo state for inclusion based on the whether they admit children routinely. We aimed to identify 10–15 hospitals for inclusion, considering this to be a feasible number of hospitals to effectively implement the project and evaluate the implementation issues involved in oxygen scale-up. After we could not identify sufficient government hospitals in Oyo state alone, we modified our criteria to include government or mission secondary health facilities (i.e. excluding private hospitals) that admitted at least 150 children per year [8]. This provided a representative sample of secondary health facilities providing hospital care for children in southwest Nigeria.

### Participant selection and consent

We used a participatory approach to select and request consent of participating hospitals. This included site visits, discussions with clinicians and administrators at individual hospitals, and a central meeting bringing together representatives from all hospitals to discuss the project in more detail. This culminated in signed agreements with each hospital to participate in the study. Hospitals were free to withdraw consent at any time.

We will not obtain consent from individual patients within the hospitals, as we are administering the intervention at the hospital level (children’s and neonatal wards), collecting de-identified data from case records without direct contact with patients and without interfering with their clinical care.. The Ethics Committee agreed that consent at the level of hospitals was appropriate. The unit of analysis will be at the individual patient level, taking into account hospital clustering. The population of interest are sick children (age 28 days to under 18 years) who are admitted to the children’s wards, and sick newborns (aged under 28 days of age) who are admitted to the neonatal ward (or require additional treatment on the maternity ward).

We will invite our project nurses, and key hospital staff at particular hospitals to participate in focus groups and/or interviews. We will obtain individual informed consent from participants of focus groups and interviews using written plain language summaries and verbal explanation in Yoruba or English (as preferred).

### Intervention

The intervention will involve the implementation of a comprehensive oxygen delivery system in the children’s and neonatal wards of 12 secondary-level hospitals in southwest Nigeria.

We used a participatory planning approach to develop the intervention and plan implementation. This involved the coordination team visiting all potential sites, conducting a mixed-methods needs assessment [8], bringing key stakeholders from each hospital together for a planning workshop, and meeting with state health authorities. Following this, the general design and components of the intervention were reviewed by revisiting the programme theory derived from past experience, results of the needs assessment, and feedback from healthcare workers, technicians, and administrators at participating hospitals. We based the intervention design on a theory-based analysis of previous oxygen projects, and used quality improvement principles, evidence-based teaching methods, and behaviour-change strategies.

Phase I involves the introduction of pulse oximeters to all participating hospitals, and the commencement of prospective data collection. The early introduction of pulse oximetry will enable accurate classification of severity of illness and indication for oxygen (which is needed for primary analysis) and will give all hospitals a tangible sign of involvement even while they are waiting for full oxygen system installation and training. During phase I, each participating hospital will be visited by the coordination team at least once to work out the site-specific approach to implementing the comprehensive oxygen system (phase II).

Phase II involves the introduction of the comprehensive oxygen system, including (1) standardised oxygen equipment package, (2) clinical education and support, (3) technical training and support, (4) procurement, installation and maintenance structures, (5) infrastructure support (including improved electricity supply using solar technology where needed), (6) strengthening disease surveillance and health information systems, and (7) strengthening quality improvement processes. This is described in detail in Table 3.

**Table 3:** ‘Improved oxygen system’ intervention components, purpose, and description

Intervention component	Purpose(s)	Description
Standardised equipment package oxygen concentrator pulse oximeter oxygen delivery equipment maintenance gear	To enable reliable, continuous access to oxygen for all children and neonates To make it easy to use oxygen correctly To make it easy to maintain oxygen equipment in good function	Selection of quality, user-friendly equipment that is proven to function in hot, humid environments and capable of being maintained with minimal technical skill: Airsep Newlife Elite oxygen concentrator, Lifebox pulse oximeter (neonatal and child probes), Airsep Sureflow flowmeter assembly, nasal prongs and tubing, oxygen analyser, installation and maintenance gear
Clinical education and support basic oximetry training healthcare worker training on the clinical use of oxygen supportive supervision	To build healthcare worker capacity and motivation to use oxygen well To stimulate healthcare workers to make their work environment more conducive to good clinical care	Clinical training material based on WHO guidelines [35, 48] and will include: clinical approaches to sick children; recognition and treatment of hypoxia; use of pulse oximetry and concentrators. Using Merrill’s approach to active learning [31], training will be active, task-based, and intentionally target motivation. Training conducted on-site at participating hospitals. One-hour basic pulse oximetry training for nurses and physicians when pulse oximeters are distributed. Half-day training on the clinical use of oxygen at the time of installation, using an ‘apprentice’ model where individual leaders are trained first, and then they are supervised to train their colleagues (and coordinate future re-training)
Technical training and support technician training on maintenance and repair supportive supervision	To build technician capacity and motivation to maintain and repair equipment To stimulate technicians to modify their procedures to make equipment care easier	Technical training material adapted from previous projects [12, 14, 15] and delivered by an expert biomedical engineer. Three-day training conducted at a central location for central engineers and at least one technician from each participating hospital. Regular supervision visits (at least 3 monthly) for re-training, review and feedback, and to identify areas needing additional attention
Procurement, installation, and maintenance structures procurement advice and support installation support maintenance procedures financing procedures	To enable reliable, continuous access to oxygen for all children and neonates To build hospital team capacity to maintain and scale up oxygen systems. To strengthen hospital’s technical capacity to maintain and repair equipment To make oxygen therapy affordable for both patients and hospitals	Uniform equipment procurement led by coordination team, in collaboration with participating hospitals Equipment installation led by central technical team in collaboration with participating hospitals, and delivered in partnership with technicians from participating hospitals Equipment procedures and forms developed by coordination team in collaboration with participating hospitals. A comprehensive maintenance plan must involve the provider, installer, engineer, local technician, and local clinical staff (including timely access to technical support) Local challenges identified during participatory planning, and addressed by local hospital teams Cost-analysis conducted by coordination team with hospital-level data, and recommendations made for action by hospital administrators
Infrastructure support improved power supply (e.g. solar power)	To ensure reliable, continuous access to oxygen for all children and neonates. To make it easy to maintain oxygen equipment in good function	Improved power system using solar capture and/or battery storage and/or generator back-up. The exact configuration has not been pre-specified, but will be based on hospital-level power evaluations, and recommendations from expert engineers/technicians (including mathematical modelling). Power system should be effective, efficient, user-friendly, and able to be maintained by local technicians. Other potential needs may include: secure storage areas, enhanced security arrangements, workspace modification, etc.
Strengthening health information systems clinical documentation medical records	To strengthen broader care processes. To strengthen managerial support for oxygen therapy.	Support nursing and medical staff to improve documentation (e.g. adapt monitoring charts to include peripheral capillary oxygen saturation (SpO_2_)) Support medical records staff with record keeping and reporting (e.g. basic electronic health reporting system on computer)
Strengthening quality improvement processes continuing education and morbidity review demonstrating quality improvement process quality improvement team building	To strengthen broader care processes To strengthen managerial support for oxygen therapy To build and sustain healthcare worker capacity and motivation to provide good clinical care To stimulate healthcare workers to make their work environment more conducive to good clinical care To build hospital team capacity to maintain and scale up oxygen systems	Support ongoing education and clinical review activities, including retraining as staff rotate Demonstrate behavioural and structural changes regarding oxygen therapy. Encourage development, and support function, of multidisciplinary teams at the hospital level Embedded project nurse within each hospital to collect data, and support project activities

Equipment manufacturers: Airsep, Buffalo, OH, USA (subsidiary of Chart Inc.); Lifebox Foundation, London, United Kingdom (http://www.lifebox.org/). We recommended the use of Airsep Newlife Elite concentrators and Lifebox pulse oximeters based on results of previous technical assessments [49–51], affordability, and field experience in resource-limited settings [10, 52]

During phase II, the coordination team will visit at least every 3 months, to provide supportive supervision, feedback, and collect user feedback. In keeping with the ideals of quality improvement, we will review ongoing user feedback and interim data to monitor implementation and enable timely response to problems in the field. Feedback to the hospitals may include written reports to congratulate them for their progress, and to inform them of how other sites are going and what the next steps in implementation may be.

Hospitals will step into phase II at different times according to pre-defined random allocation. As such, hospitals will be in phase I for between 4 and 16 months, and in phase II for between 8 and 20 months, with a total period of 24 months of prospective data collection.

### Outcome measures

To achieve our primary objective of understanding how to most effectively implement oxygen systems, our mixed-methods evaluation will assess effectiveness, process and fidelity and measure a range of clinical, quality of care, technical, and health systems outcomes (including cost, sustainability, and managerial/policy implications) (Table 4).

**Table 4:** Outcome measures used to evaluate the Nigeria Oxygen Implementation Project with associated research question and data source

Category	Research question(s)	Outcome measures	Data source
Clinical effectiveness	Does the intervention improve outcomes for the target population (specifically child pneumonia, pre-term/small neonates)?	Under-five case fatality rate (all-cause) Under-five pneumonia case fatality rate Neonatal case fatality rate Pre-term/small neonatal case fatality rate	Case notes
Epidemiological	What are the characteristics of patients with hypoxaemia in these hospitals?	Mean duration of hypoxaemia, oxygen therapy Descriptive statistics regarding hypoxaemia and oxygen therapy (age, condition, illness severity, etc.)	Case notes
Quality of care – oxygen practices	Can healthcare works correctly identify, treat, and monitor children and neonates who need oxygen therapy? Does the intervention improve healthcare workers’ oxygen care practices? How is the intervention adopted by healthcare workers? What training and supervisions is required to change practice?	Proportion of hypoxaemic children who correctly received oxygen therapy Proportion of admitted children who have pulse oximetry done correctly during admission Proportion of children receiving oxygen who had a documented clinical indication for oxygen Mean quality of care score for oxygen therapy Mean knowledge/skill scores Attitudes, behaviours, and feedback from healthcare workers (qualitative)	Case notes Knowledge/skill test Interviews/focus groups
Quality of care – broader care	What is the quality of inpatient paediatric hospital care? Does the intervention improve healthcare workers’ broader care practices?	Mean quality of care score for target conditions (pneumonia, malaria, pre-term/small neonate) Proportion of admitted children discharged against medical advice Mean length of stay Healthcare worker motivation, satisfaction Structural and process determinants of care quality (medical supplies, hygiene, guidelines, clinical review meetings, record keeping, etc.)	Case notes Interviews
Technical	Can hospital staff reliably maintain the oxygen equipment in working order? What technical problems are reported, how common, what solution, what cost?	Proportion of oxygen concentrators clean and in working order (producing > 85% oxygen, adequate flow, etc.) Proportion of pulse oximeters clean and in working order Proportion of time when concentrators were not functional, or not available for use (and reasons for non-availability) Proportion of solar power (or other improved power) systems in working order (and reasons for failure) Proportion of maintenance checks actually completed Descriptive statistics regarding technical problems identified, solutions, and costs	Technician log book (includes Standardised Report Form)
Fidelity	Was the intervention implemented as planned?	Actual timing of intervention vs intended (including training, installation, and supervision activities) Actual composition of intervention vs intended (including training, installation, and supervision activities)	Administrative records Technician log book
Cost	What is the cost of the intervention?	Equipment and installation costs Training and supervision costs Mean cost per life saved Mean cost per disability-adjusted life year (DALY) saved	Administrative records Case notes
Managerial and policy	Can the intervention be integrated in hospital managerial and state policy structures?	Hospital implementation of oxygen financing recommendations Hospital implementation of ‘oxygen team’ structure State commitment and funding for oxygen programme National impact on oxygen policy and guidelines AFASS (Acceptability, Feasibility, Affordability, Sustainability, Safety) Unintended effects Barriers and enablers	Administrative records Interviews
Sustainability	Can the intervention be sustained in the medium to long term?	Assess at 1, 2 and 5 years (using indicators above): • Technical function and maintenance processes • Healthcare worker oxygen practices • Cost recovery and ongoing financing • Hospital-level scale-up • Managerial and policy integration and scale-up	Technician log book Interviews

Case fatality rate = proportion of admitted population that die in hospital or are discharged unwell expected to die. Quality of care composite measures derived from WHO hospital care for children guidelines

### Clinical outcomes

We will collect data on clinical outcomes from case notes to evaluate clinical effectiveness and to enable exploration of variability of effect between hospitals. The primary clinical outcome will be in-hospital case fatality rates for the target population. Secondary clinical outcomes will include a range of quality of care measures relating to oxygen therapy, and supportive care. We will assess quality of care based on key clinical activities at various stages of care (assessment, diagnosis/classification, management, monitoring) based on WHO guidelines for the hospital care for children [35]. This approach is similar to the recently described Paediatric Admission Quality of Care (PAQC) score [36].

### Implementation outcomes

Our broader approach for evaluating the Nigeria Oxygen Implementation Project is guided by recommendations on the evaluation of complex interventions, particularly the theory-based realist evaluation approach [37–43]. Our programme theory articulates a series of ‘mechanisms’ through which particular parts of our intervention will impact on clinical outcomes. As such, our evaluation seeks to answer three broad questions. First, was the intervention implemented as we intended? Second, did the intervention affect the identified mechanism as we expected? Third, did the mechanisms activated by the intervention result in change clinical outcomes?

We will collect quantitative and qualitative data to answer these implementation questions. The outcome measures are described in Table 4, together with the corresponding research question and data source. The timeline of data collection is illustrated in Fig. 1.

### Sample size

We made sample size and power estimates using the Stata menu-driven programme *steppedwedge*, which is based on the Hussey and Hughes’ method for calculating sample size for a stepped-wedge trial [44, 45]. We based our assumptions on admission, prevalence, and case-fatality rate data from our needs-analysis, and informed by correlation estimates from previous studies [46]. We estimated that we would enrol approximately 16,770 children and 7670 neonates (1290 preterm) over the course of the study. Based on an alpha of 5%, we calculated that we would have approximately 80% power to detect a 35% reduction in pneumonia case fatality rate (6.1% to 4%), and 90% power to detect a 20% reduction in neonatal case fatality rate (8.2% to 6.6%). We would have greater than 90% power to detect 10% change in quality of care scores.

### Allocation

We used pre-randomisation stratification to ensure each step included one ‘large’ (>1500 child admissions per year) and two ‘small/medium’ hospitals (<1500 admissions), as we supposed that the size of hospital (and associated staffing and expertise) may influence the outcomes. We randomised hospitals to receive the intervention in steps 2, 3, 4, or 5, using a computer-generated random sequence generator, resulting in one ‘large’ and two ‘small’ hospitals allocated to receive the intervention at equally spaced 4-month intervals. This stepped-wedge randomised design enabled all hospitals to receive the intervention, while maintaining a robust controlled design (individual hospitals acting as their own historical control, and each other’s contemporaneous control).

We conducted randomisation with four members of the research team in attendance, and shared the randomisation order with hospitals as part of the participatory planning process. It was not possible to blind patients, healthcare-providers, or hospital administrators from the intervention due to the nature of the intervention.

### Data management and analysis

Clinical data will be collected from individual case notes by trained nurse data collectors. These data collectors will complete standardised Case Report Forms (CRF1) (see Additional file 2), which will be collated and returned to the project manager before being handed to the data manager. Trained data-entry clerks will double-enter the data into EpiData (EpiData Association, Odense, Denmark). The data manager will supervise the data entry and verification process, and will export the raw data file into.dta format for analysis using Stata v14 (StataCorp, College Station, TX, USA). All data will be de-identified before analysis to preserve confidentiality. The primary investigators will have full access to the data, and summary data will be made available for external validation purposes.

We will report characteristics of the individuals and clusters descriptively to illustrate potential imbalance between exposed and unexposed and allow for discussion of potential biases. We will illustrate the number of observations within each cluster by overlaying the summary numbers onto a stepped-wedge diagram. We will analyse effectiveness using intention-to-treat, according to the time hospitals were randomised to crossover. We will report any differences between intended crossover time and actual crossover time. We will adjust for the effect of calendar time, clustering, and other significant confounders using generalised linear mixed models (GLMM) or generalised estimating equations (GEE) [32, 44]. We will examine heterogeneity in effects between different hospitals using within-cluster comparisons of exposed and unexposed periods. This will contribute to better understanding of how various contextual factors influence effectiveness. We will conduct 6-monthly reviews of data for presentation to a supervisory committee, and to ensure that there is no evidence of significant unintended harm. Given the anticipated benefits and safety of this intervention, we do not have a formal data monitoring committee.

Equipment data will be collected by trained biomedical technicians during routine preventive maintenance visits. These technicians will complete standardised Case Report Forms (CRF2), and return them to the project manager. They will be entered and verified using the same procedures as for CRF1. We will report equipment outcome data descriptively, allowing for comparison between different hospitals.

Healthcare worker knowledge will be assessed using individual written tests (TEST) before and after each training, and at 6-month periods post implementation. Trainers will administer these tests, collate the completed tests, and return them to the project manager. They will be entered and verified using the same procedures as for CRF1. We will report before and after test scores in relation to the basic pulse oximetry training, full oxygen training, and at follow-up. We will report confidence intervals for all estimates, and assess for statistical significance using a *t* test for (normally distributed) continuous outcomes, and chi-squared for binary outcomes.

Qualitative data from interviews and focus groups will be transcribed and verified for accuracy by participants. We will import these to NVivo v11 (QSR International, Doncaster, VIC, Australia) for data management and analysis. We will use the qualitative data to explore the reasons behind the observed intervention effects (the ‘mechanisms’) and variation between hospitals (the contextual influences). To achieve this we will use a Grounded Theory approach to data analysis, using mixed inductive and deductive content analysis approaches.

Other process data will be collected from administrative records, finance ledgers, and correspondence between investigators and hospitals. We will use this data to understand how the intervention was implemented, identify new avenues of inquiry, and collate practical lessons for future application.

We will report results using the CONSORT 2010 extension to cluster randomised trials, with modifications for the stepped-wedge design as proposed by Hemming et al. [32]. We will publish our results in an open-access journal.

### Ethics

This study obtained ethics approval from the University of Melbourne (1543797.1) and University of Ibadan/University College Hospital Ethics Committee, Ibadan, Nigeria (UI/EC/16/0413). We registered the trial on the Australian and New Zealand Clinical Trials Registry (ACTRN12617000341325). Data monitoring will be done by the lead author, reporting to an advisory committee every 6 months.

## Discussion

This project is part of a multi-country implementation research project based in Nigeria and Papua New Guinea [47], which seeks to test and build upon the implementation lessons learnt from oxygen projects in Papua New Guinea [9, 14] and elsewhere [10]. By using a realist approach to project design and evaluation, this project will provide information not only on *what* works, but *how* it works in different contexts. We consider this information critical to meaningfully scale up oxygen therapy globally and ensure that this basic medical therapy is available to those who need it the most.

Two previous oxygen projects have demonstrated that improved oxygen systems can improve paediatric pneumonia outcomes for hospitalised children [9, 15]. This project will advance the evidence on effectiveness using a rigorous, randomised control design to evaluate clinical outcomes in admitted children and neonates in a challenging sub-Saharan African setting.

At the time of writing we have commenced activities in all 12 participating hospitals. We completed a needs assessment and participatory planning process in 2015, and implemented the full oxygen system in the first three hospitals in April 2016. The final three hospitals have commenced intervention activities in March 2017, as scheduled. The main implementation challenges encountered so far have related to the development and testing of the solar power systems, necessitating a complete redesign and pilot testing of the system. We took alternative measures in the interim to improve and stabilise the power systems for the early intervention hospitals and were able to complete full implementation of the improved oxygen systems according to the planned stepped-wedge timeline.

As expected, different hospitals have taken slightly different approaches to particular aspects of the intervention. For example, while hospital administrators have been open to alternative financing structures for oxygen, the actual implementation of this has been difficult in some hospitals due to administrative and governance requirements. We have been impressed with the ingenuity and problem-solving capacity of the participating hospitals, but informal feedback shows significant variability in the functioning of teams at different hospitals. This highlights the importance of measuring and reporting fidelity and other process indicators, in order to better understand this variability and how improved oxygen systems work in different contexts. Overall, the intervention appears to be achieving very high levels of fidelity, and early data shows large improvements in oxygen-related clinical behaviours (e.g. the use of pulse oximetry, administration of oxygen to hypoxaemic patients, etc.). Interim safety data suggests that the intervention is not causing harm and probably causing benefit, and we anticipate having adequate power to detect a meaningful difference by the end of data collection despite lower than expected admission numbers.

Oxygen therapy is a basic, life-saving therapy that is relevant for many paediatric and adult health conditions, but has limited global availability. Our study should provide quality evidence on the effectiveness of improved oxygen systems, and how to better implement and scale up oxygen systems in resource-limited settings. Our results will have important implications for policy-makers, hospital administrators, and child health organisations in Africa and globally. Our findings may have broader applicability to other healthcare interventions that are worthy of scale-up but are proving challenging to implement in the places that can benefit the most.

### Trial status

This trial is underway. Recruitment, clinical data collection, project team visits, and interviews are all ongoing.

## Additional files

Additional file 1: SPIRIT Checklist. (DOC 121 kb)

Additional file 2: Data Collection Forms. (ZIP 2651 kb)

## Abbreviations

BCW: Behaviour Change Wheel
GEE: Generalised estimating equations
GLMM: Generalised linear mixed model
PAQC: Paediatric Admission Quality of Care
SpO_2_: Blood oxygen saturation measured by pulse oximetry
TDF: Theoretical Domains Framework
U/5: Under 5 years of age
WHO: World Health Organisation
