# Supplementary material for: Improving oxygen therapy for children and neonates in secondary hospitals in Nigeria: study protocol for a stepped-wedge cluster randomised trial
Source: Trials. 2017 Oct 27;18:502. doi: 10.1186/s13063-017-2241-8 (PMC5659007; doi:10.1186/s13063-017-2241-8)
Supplement: Supplementary file 2 — Data Collection Forms. (ZIP 2651 kb) [file 13063_2017_2241_MOESM2_ESM.zip › CRF1_CHILD_FINALR1.pdf]

CRF 1 (child): Children admitted to Hospital

Hospital No: ________________________ **Serial No:** __ __ __ __ **Nurse ID:** __ __

State ID: __ **Health Facility ID:** __ __ **Study ID:** __ /__ __ /__ __ __ __ /__

INSTRUCTIONS
**Specific instructions for CRF1 (child)**<br>• *CFR1 (child)* must be completed for every child admitted to hospital. *CFR1 (child)* It is not for use with Pre-term or other Neonates (aged <30 days) [see *CFR 1 (neon)*] or for Children seen in outpatients but not admitted.<br>• The name of the patient should not appear on the *CRF* (confidentiality).
**General instructions**<br>• Please use a black or blue ballpoint pen. Answer every question.<br>• Print all written entries with BLOCK CAPITAL LETTERS.<br>• Mark boxes with a cross (X) where requested (e.g. ☒).<br>• All date entries must appear in the format dd/mm/yyyy (e.g. 23/09/2015).<br>• If exact time is not available, estimate to the nearest hour.<br>• If you make an error - draw a line through the error and write the correct value next to it. Date and initial the correction.

PART A – Nurse's Admission Form

This form should have been completed by the Admitting Nurse at the time of admission, and inserted in the Case Notes. It will be done only at particular times. Please collect the completed form from the Case Notes and attach/copy it to this CRF.

0	Was the Nurse's Admission Form completed and available in the Case notes?	☐1 YES => attach/copy below, then continue to Part B<br>☐2 NO => continue to Part B

1	On admission, did the child have:	Cool hands and feet	☐1 YES ☐2 NO
		Very active or not cooperative	☐1 YES ☐2 NO
		Very agitated or upset	☐1 YES ☐2 NO
		Shivering	☐1 YES ☐2 NO
		Oedema of hands or feet	☐1 YES ☐2 NO
		Painted nails (e.g. henna, nail polish)	☐1 YES ☐2 NO
2	Was pulse oximetry attempted?	☐ **YES**<br>- How many times was it attempted (before you got a good reading or you gave up)?<br>☐1 Once (1)<br>☐2 Twice (2)<br>☐3 Three or more (3+)<br>*(Continue to Q3)*	☐ **NO** - Why not?<br>☐4 Oximeter was not on the ward<br>☐5 Oximeter was being used for someone else<br>☐6 Oximeter was broken or not working properly<br>☐7 Sensor probe was broken<br>☐8 Child was too sick<br>☐9 Other (please specify):<br>*(The End)*
3	Was a successful reading obtained on pulse oximetry?	☐1 **YES**	☐ **NO** - What was the problem?<br>☐2 Could not get a good trace<br>☐3 Sensor probe did not fit<br>☐4 Other (please specify):
4	What was the SpO2?	__________%	Not successful ☐0
5	Did the oximeter show a regular pleth waveform?	☐1 YES ☐2 NO	I don't know / Not done ☐0
6	Did the oximeter heart rate indicator match the child's pulse on palpation?	☐1 YES ☐2 NO	I don't know / Not done ☐0
7	Estimate how many minutes were spent doing pulse oximetry?	_____________ minutes	

CRF 1 (child): Children admitted to Hospital

Hospital No: ________________________ Serial No: __ __ __ __ Nurse ID: __ __

State ID: __ Health Facility ID: __ __ Study ID: __ /__ __/__ __ __ __ /__

PART B – General Admission Details			
8	Hospital Name		
9	Hospital Patient ID	__ __ __ __ __ __ __ __ __ __	
10	Date of birth	__ __ / __ __ / __ __ __ __ (dd/mm/yyyy) Unknown ☐99	
11	Age	_______ Years, _______ Months	
12	Sex	☐1 Male ☐2 Female	
13	Date of Admission	__ __ / __ __ / __ __ __ __ (dd/mm/yyyy)	
14	Referred FROM	☐1 Home Unknown ☐99<br>☐2 Primary Health Centre (PHC)<br>☐3 Secondary Health Centre<br>(e.g. General or State hospital)<br>☐4 Mission Hospital<br>☐5 Private Hospital / Clinic<br>☐6 Traditional Health Care Provider<br>☐ Other (specify): _____________	
15	Date of Discharge/death/abscond/transfer	__ __ / __ __ / __ __ __ __ (dd/mm/yyyy)	
16	Outcome	☐1 Discharged well<br>☐2 Died in hospital<br>☐3 Discharged unwell, recovery not expected<br>☐4 Discharged against medical advice (DAMA)<br>☐5 Absconded<br>☐6 Transferred to other hospital (specify): _____________	
17	Primary <u>admission</u> diagnosis<br>(main reason for admission recorded in the doctor's admission note)	1. __ __ None recorded ☐99<br>Other (specify): _____________	*See **Diagnosis Codes** on separate page*
18	Other <u>admission</u> diagnoses<br>(any additional diagnoses recorded in the doctor's admission note)	1. __ __ None recorded ☐99<br>2. __ __<br>3. __ __<br>4. __ __<br>Other (specify): _____________	
19	Primary <u>discharge</u> diagnosis<br>(main reason for admission recorded in the doctor's final case note)	1. __ __ None recorded ☐99<br>Other (specify): _____________	
20	Other <u>discharge</u> diagnoses<br>(any additional diagnoses recorded in the doctor's final case note)	1. __ __ None recorded ☐99<br>2. __ __<br>3. __ __<br>4. __ __<br>Other (specify): _____________	

CRF 1 (child): Children admitted to Hospital

Hospital No: ______________________ Serial No: __ __ __ __ Nurse ID: __ __

State ID: __ Health Facility ID: __ __ Study ID: __ /__ __/__ __ __ __ /__

**PART C - Oxygen therapy**		
**24**	Was SpO2 less than 90% (SpO2 <90%) at any time during admission?	☐1 YES ☐2 NO Not recorded ☐99
**25**	Was oxygen therapy given at any time during admission?	☐1 YES<br>☐2 NO => go to **PART D**
Starting oxygen		
**26**	Date that oxygen was <u>first started</u>*	__ __ / __ __ / __ __ __ __ (dd/mm/yyyy)
**27**	Last Sp02 recorded <u>before</u> oxygen was first started*	__ __ __% Not recorded ☐99
**28**	Flow rate when oxygen was first started*	__ L/min Not recorded ☐99
Stopping oxygen		
**29**	Date that oxygen was <u>finally ceased</u>*	__ __ / __ __ / __ __ __ __ (dd/mm/yyyy)<br>Oxygen was not ceased# ☐88<br>Not recorded ☐99
30	Last Sp02 recorded <u>before</u> oxygen was finally ceased*	__ __ __% Not recorded ☐99
31	First Sp02 recorded <u>after</u> oxygen was finally ceased*	__ __ __% Not recorded ☐99
32	What was the last Sp02 prior to <u>discharge/death/referral</u>?	__ __ __% Not recorded ☐99
Monitoring oxygen saturations (SpO2) while <u>on oxygen</u>		
**33**	While the child was on oxygen - were Sp02 readings recorded at least twice per day?	☐1 YES ☐2 NO
**34**	While the child was on oxygen - how many Sp02 readings **<85%**?	__ __
**35**	While the child was on oxygen - how many Sp02 readings **86-90%**?	__ __
**36**	While the child was on oxygen - how many Sp02 readings **91-95%**?	__ __
**37**	While the child was on oxygen - how many Sp02 readings **96-100%**?	__ __

* Oxygen may have been started and stopped multiple times during admission. "First started" means when the child when on oxygen for the first time. "Finally ceased" means when the child had oxygen stopped for the final time.

# "Oxygen was not ceased" means if the patient died, absconded, DAMA or was referred while still on oxygen.

Hospital No: ______________________ Serial No: __ __ __ __ Nurse ID: __ __

State ID: __ Health Facility ID: __ __ Study ID: __ /__ __/__ __ __ __ /__

PART D – Findings on Admission, Case definitions

**Signs and Symptoms (at Admission)**			
**35**	History of fever	☐1 YES ☐2 NO	Not recorded ☐99
**36**	Cough or difficult breathing	☐1 YES ☐2 NO	Not recorded ☐99
**37**	Respiratory distress<br>(e.g. grunting, gasping, severe chest indrawing)	☐1 YES ☐2 NO	Not recorded ☐99
**38**	Central cyanosis (blue lips or tongue)	☐1 YES ☐2 NO	Not recorded ☐99
**39**	Pallor (pale conjunctiva or skin)	☐1 YES ☐2 NO	Not recorded ☐99
**40**	Jaundice (yellow eyes or skin)	☐1 YES ☐2 NO	Not recorded ☐99
**41**	Diarrhoea (>3 per day)	☐1 YES ☐2 NO	Not recorded ☐99
**42**	Unable to breastfeed or drink adequately	☐1 YES ☐2 NO	Not recorded ☐99
**43**	Convulsions (seizures, fits)	☐1 YES ☐2 NO	Not recorded ☐99
**44**	Confusion or lethargy (difficult to wake)	☐1 YES ☐2 NO	Not recorded ☐99
**45**	Coma (unconscious or barely conscious)	☐1 YES ☐2 NO	Not recorded ☐99
**46**	Signs of shock (cold hands, capillary refill > 3 secs, high heart rate with weak pulse, low or unmeasurable blood pressure)	☐1 YES ☐2 NO	Not recorded ☐99
**47**	Signs of severe dehydration<br>(lethargy, sunken eyes, decreased skin turgor)	☐1 YES ☐2 NO	Not recorded ☐99
**Measurements and Investigations (at Admission)**			
**48**	Weight	__ __ . __ kg	Not recorded ☐99
**49**	Height / length	__ __ __ cm	Not recorded ☐99
**50**	Heart rate (HR)	__ __ __ bpm	Not recorded ☐99
**51**	Respiratory rate (RR)	__ __ cpm	Not recorded ☐99
**52**	SpO2 on pulse oximetry (%)	__ __ __ %	Not recorded ☐99
**53**	Temperature (Celsius)	__ __ . __ Celsius	Not recorded ☐99
**54**	PCV (Packed Cell Volume)	__ __ %	Not recorded ☐99
**55**	Malaria Rapid Diagnostic Test (RDT)	☐1 Positive ☐2 Negative	Not recorded ☐99
**56**	Malaria blood film (microscopy)	☐1 Positive ☐2 Negative	Not recorded ☐99
**57**	HIV Test	☐1 Positive ☐2 Negative	Not recorded ☐99
**58**	Did the child have Malnutrition? (weight-for-length <-3SD, OR MUAC <115mm, OR oedema of both feet)	☐1 YES ☐2 NO	Not recorded ☐99

**Case definitions** – if the child fits more than one case definition, complete all the relevant Parts		
**59**	Did the child have <u>Cough or difficult breathing</u> **AND** any of the following signs:<br>• Fast breathing (age 2-11 months, ≥50cpm; age 1-5 years, ≥40cpm)<br>• Lower chest wall indrawing (respiratory distress)	☐1 YES ☐2 NO<br>=> if Yes, **<u>PART E - Pneumonia</u>** must be completed.
**60**	Did the child have <u>History of Fever/Temp >37.5 C</u> **AND** <u>Positive Malaria test</u> (blood film or rapid diagnostic test)	☐1 YES ☐2 NO<br>=> if Yes, **<u>PART F - Malaria</u>** must be completed.
**61**	Did the child have watery <u>Diarrhoea</u> (>3 per day) that is not for >14 days duration?	☐1 YES ☐2 NO<br>=> if Yes, **<u>PART G - Diarrhoea</u>** must be completed.

CRF 1 (child): Children admitted to Hospital

Hospital No: ________________________ Serial No: __ __ __ __ Nurse ID: __ __

State ID: __ Health Facility ID: __ __ Study ID: __ /__ __/__ __ __ __ /__

PART E – Pneumonia

62	Did the Admission Diagnosis indicate Severe Pneumonia or Pneumonia?	☐1 Pneumonia<br>☐2 Severe pneumonia	Not recorded ☐99
63	Duration of cough	__ __ days	Not recorded ☐99
64	Was a wheeze found on auscultation (chest exam)?	☐1 YES ☐2 NO	Not recorded ☐99
65	Was a chest x-ray (CXR) done?<br>(if yes, specify result)	☐1 YES ☐2 NO	Not recorded ☐99
66	Was tuberculosis (TB) testing done<br>(e.g. TST/Mantoux, GeneXpert)?<br>(if yes, specify result)	☐1 YES ☐2 NO	Not recorded ☐99
67	Was oral feeding stopped?	☐1 YES ☐2 NO	Not recorded ☐99
68	Were intravenous (IV), intramuscular (IM), or oral antibiotics given on the first day of admission?	☐1 Intravenous (IV)<br>☐2 Intramuscular (IM)<br>☐3 Oral	None / Not recorded ☐99<br>**=> go to Q70**
69	What antibiotics were given on the first day of admission? What dose, frequency, and duration?<br>(complete all that apply)<br>*See **Antibiotic codes** on separate page*<br><br>*Fill the corresponding **drug dose** in the table*	1 Drug: __ __<br>Frequency: __ times per day<br>Duration: __ __ days<br>2 Drug: __ __<br>Frequency: __ times per day<br>Duration: __ __ days<br>3 Drug: __ __<br>Frequency: __ times per day<br>Duration: __ __ days	 Not recorded ☐99<br>Not recorded ☐99<br><br>Not recorded ☐99<br>Not recorded ☐99<br><br>Not recorded ☐99<br>Not recorded ☐99

	DOSE			
**DRUGS**	mg	ml	tab	mega unit
1				
2				
3				

70	Were intravenous (IV) or nasogastric (NG) fluids started on the first day of admission?	☐1 YES ☐2 NO **=> go to Q73**	
71	What intravenous (IV) fluids were given on the first day of admission, at what rate were they started, and for what duration?<br>*See **IV fluid codes** on separate page*	1 Fluid type: __ __<br>Rate: __ __ __ ml/hour<br>Duration: __ __ hours<br>2 Fluid type: __ __<br>Rate: __ __ __ ml/hour<br>Duration: __ __ hours	Not recorded ☐99<br>Not recorded ☐99<br>Not recorded ☐99<br>Not recorded ☐99<br>Not recorded ☐99<br>Not recorded ☐99
72	What nasogastric (NG) fluids were given on the first day of admission, at what rate were they started, and for what duration?<br>*See **NG fluid codes** on separate page*	Fluid type: __ __<br>Rate: __ __ __ ml/hour<br>Duration: __ __ hours	Not recorded ☐99<br>Not recorded ☐99<br>Not recorded ☐99
73	How many times were nursing observations documented per day during the first 3 days of admission? (e.g. respiratory rate, heart rate)	Day 1: __ __ times per day<br>Day 2: __ __ times per day<br>Day 3: __ __ times per day	Not recorded ☐99<br>Not recorded ☐99<br>Not recorded ☐99

Hospital No: ______________________ Serial No: __ __ __ __ Nurse ID: __ __

State ID: __ Health Facility ID: __ __ Study ID: __ /__ __/__ __ __ __ /__

PART F – Malaria

No	Question	Response	
74	Did the Admission Diagnosis indicate Severe Malaria or Malaria?	☐1 Malaria<br>☐2 Severe malaria	Not recorded ☐99
75	Was neck stiffness and rash present?	☐1 YES ☐2 NO	Not recorded ☐99
76	Signs of infection on Urine testing?	☐1 YES ☐2 NO	Not recorded ☐99
77	Signs of infection on lumbar puncture (CSF)?	☐1 YES ☐2 NO	Not recorded ☐99
78	Was a chest x-ray (CXR) done?<br>(if yes, specify result)	☐1 YES ☐2 NO	Not recorded ☐99
79	Was Blood Sugar testing done?	☐1 YES ☐2 NO	Not recorded ☐99
80	Was a blood transfusion given?	☐1 YES ☐2 NO	Not recorded ☐99
81	Was iron-folate supplement given?	☐1 YES ☐2 NO	Not recorded ☐99
82	Were intravenous (IV), intramuscular (IM), or oral <u>anti-malarials given on the first day of admission</u>?	☐1 Intravenous (IV)<br>☐2 Intramuscular (IM)<br>☐3 Oral	None / Not recorded ☐99<br>**=> go to Q84**
83	What anti-malarial drugs were given, what dose, frequency, and duration?<br>(complete all that apply)<br>*See **Antimalarial codes** on separate page*<br>*Fill the corresponding **drug dose** in the table*	1 Drug: __ __<br>Frequency: __ times per day<br>Duration: __ __ days<br>2 Drug: __ __<br>Frequency: __ times per day<br>Duration: __ __ days<br>3 Drug: __ __<br>Frequency: __ times per day<br>Duration: __ __ days	 Not recorded ☐99<br>Not recorded ☐99<br><br>Not recorded ☐99<br>Not recorded ☐99<br><br>Not recorded ☐99<br>Not recorded ☐99

	DOSE		
**DRUGS**	mg	ml	tab
1			
2			
3			

No	Question	Response	
84	Were intravenous (IV) or nasogastric (NG) fluids started on the <u>first day of admission</u>?	☐1 YES ☐2 NO / Not recorded **=> go to Q87**	
85	What <u>intravenous (IV)</u> fluids were given, at what rate were they started, and for what duration?<br>*See **IV fluid codes** on separate page*	1 Fluid type: __ __<br>Rate: __ __ __ ml/hour<br>Duration: __ __ hours<br>2 Fluid type: __ __<br>Rate: __ __ __ ml/hour<br>Duration: __ __ hours	Not recorded ☐99<br>Not recorded ☐99<br>Not recorded ☐99<br>Not recorded ☐99<br>Not recorded ☐99<br>Not recorded ☐99
86	What <u>nasogastric (NG)</u> fluids were given, at what rate were they started, and for what duration?<br>*See **NG fluid codes** on separate page*	Fluid type: __ __<br>Rate: __ __ __ ml/hour<br>Duration: __ __ hours	Not recorded ☐99<br>Not recorded ☐99<br>Not recorded ☐99
87	How many times were nursing observations documented per day during the first 3 days of admission? (e.g. respiratory rate, heart rate)	Day 1: __ __ times per day<br>Day 2: __ __ times per day<br>Day 3: __ __ times per day	Not recorded ☐99<br>Not recorded ☐99<br>Not recorded ☐99

CRF 1 (child): Children admitted to Hospital

Hospital No: ________________________ Serial No: __ __ __ __ Nurse ID: __ __

State ID: __ Health Facility ID: __ __ Study ID: __ /__ __/__ __ __ __ /__

PART G – Diarrhoea		
88	Did the Admission Diagnosis indicate the severity of Dehydration?	☐0 No dehydration<br>☐1 Some dehydration<br>☐2 Severe dehydration<br>Not recorded ☐99
89	What was the duration of diarrhoea?	__ __ days<br>Not recorded ☐99
90	What was the frequency of diarrhoea (per day)?	__ __ per day<br>Not recorded ☐99
91	Was there bloody diarrhoea?	☐1 YES ☐2 NO Not recorded ☐99
92	Was there persistent vomiting?	☐1 YES ☐2 NO Not recorded ☐99
93	Was Blood Sugar testing done?	☐1 YES ☐2 NO Not recorded ☐99
94	Was oral feeding <u>stopped</u>?	☐1 YES ☐2 NO Not recorded ☐99
95	Was zinc supplement started?	☐1 YES ☐2 NO Not recorded ☐99
96	Were antibiotics given?	☐1 YES ☐2 NO / Not recorded **=> go to Q98**
97	What antibiotics were given, what dose, frequency, and duration?<br>(complete all that apply)<br>*See **Antibiotic codes** on separate page*<br><br>*Fill the corresponding **drug dose** in the table*	1 Drug: __ __<br>Frequency: __ times per day Not recorded ☐99<br>Duration: __ __ days Not recorded ☐99<br>2 Drug: __ __<br>Frequency: __ times per day Not recorded ☐99<br>Duration: __ __ days Not recorded ☐99<br>3 Drug: __ __<br>Frequency: __ times per day Not recorded ☐99<br>Duration: __ __ days Not recorded ☐99

	DOSE			
**DRUGS**	mg	ml	tab	mega unit
1				
2				
3				

98	Were intravenous (IV) or nasogastric (NG) fluids started on the <u>first day of admission</u>?	☐1 YES ☐2 NO / Not recorded **=> go to Q101**
99	What <u>intravenous (IV)</u> fluids were given, at what rate were they started, and for what duration?<br>*See **IV fluid codes** on separate page*	1 Fluid type: __ __ Not recorded ☐99<br>Rate: __ __ __ ml/hour Not recorded ☐99<br>Duration: __ __ hours Not recorded ☐99<br>2 Fluid type: __ __ Not recorded ☐99<br>Rate: __ __ __ ml/hour Not recorded ☐99<br>Duration: __ __ hours Not recorded ☐99
100	What <u>nasogastric (NG)</u> fluids were given, at what rate were they started, and for what duration?<br>*See **NG fluid codes** on separate page*	Fluid type: __ __ Not recorded ☐99<br>Rate: __ __ __ ml/hour Not recorded ☐99<br>Duration: __ __ hours Not recorded ☐99
101	How many times were nursing observations documented per day during the first 3 days of admission? (e.g. respiratory rate, heart rate)	Day 1: __ __ times per day Not recorded ☐99<br>Day 2: __ __ times per day Not recorded ☐99<br>Day 3: __ __ times per day Not recorded ☐99
