# Supplementary material for: Improving oxygen therapy for children and neonates in secondary hospitals in Nigeria: study protocol for a stepped-wedge cluster randomised trial
Source: Trials. 2017 Oct 27;18:502. doi: 10.1186/s13063-017-2241-8 (PMC5659007; doi:10.1186/s13063-017-2241-8)
Supplement: Supplementary file 2 — Data Collection Forms. (ZIP 2651 kb) [file 13063_2017_2241_MOESM2_ESM.zip › CRF1_NEONATE_FINALR1.pdf]

CRF 1 (neonate): Neonates admitted to SCBU

Hospital No: ______________________ Serial No: __ __ __ __ Nurse ID: __ __

State ID: __ Health Facility ID: __ __ Study ID: __/__ __/__ __ __ __/__

INSTRUCTIONS
**Specific instructions for CRF1 (neonate)**<br>• *CRF1 (neonate)* must be completed for <u>every neonate admitted to SCBU</u>. This includes: newborns that have been transferred from another hospital ('outborn'); newborns born in this hospital ('inborn') AND require more than routine postnatal care; babies <28 days of age that are admitted to hospital. *CRF1 (neonate)* is <u>not</u> for newborns who are well and require routine postnatal care only.<br>• The name of the patient should <u>not</u> appear on the *CRF* (confidentiality).
**General instructions**<br>• Please use a black or blue ballpoint pen. Answer every question.<br>• Print all written entries with BLOCK CAPITAL LETTERS.<br>• Mark boxes with a cross (X) where requested (e.g. ☒).<br>• All date entries must appear in the format dd/mm/yyyy (e.g. 23/09/2015).<br>• If exact time is not available, estimate to the nearest hour.<br>• If you make an error - draw a line through the error and write the correct value next to it. Date and initial the correction.

PART A – Nurse's Admission Form

This form should have been completed by the Admitting Nurse at the time of admission, and inserted in the Case Notes. It will be done only at particular times. Please collect the completed form from the Case Notes and attach/copy it to this CRF.

**0**	Was the Nurse's Admission Form completed and available in the Case notes?	☐1 YES => attach/copy below, then continue to Part B<br>☐2 NO => continue to Part B

1	On admission, did the child have:	Cool hands and feet	☐1 YES ☐2 NO
		Very active or not cooperative	☐1 YES ☐2 NO
		Very agitated or upset	☐1 YES ☐2 NO
		Shivering	☐1 YES ☐2 NO
		Oedema of hands or feet	☐1 YES ☐2 NO
		Painted nails (e.g. henna, nail polish)	☐1 YES ☐2 NO
2	Was pulse oximetry <u>attempted</u>?	☐ **YES**<br>- How many times was it attempted (before you got a good reading or you gave up)?<br>☐1 Once (1)<br>☐2 Twice (2)<br>☐3 Three or more (3+)<br>*(Continue to Q3)*	☐ **NO** - Why not?<br>☐4 Oximeter was not on the ward<br>☐5 Oximeter was being used for someone else<br>☐6 Oximeter was broken or not working properly<br>☐7 Sensor probe was broken<br>☐8 Child was too sick<br>☐9 Other (please specify):<br>*(The End)*
3	Was a <u>successful reading</u> obtained on pulse oximetry?	☐1 **YES**	☐ **NO** - What was the problem?<br>☐2 Could not get a good trace<br>☐3 Sensor probe did not fit<br>☐4 Other (please specify):
4	What was the SpO2?	__________%	Not successful ☐0
5	Did the oximeter show a regular <u>pleth waveform</u>?	☐1 YES ☐2 NO	I don't know / Not done ☐0
6	Did the oximeter <u>heart rate indicator</u> match the child's pulse on palpation?	☐1 YES ☐2 NO	I don't know / Not done ☐0
7	Estimate how many minutes were spent doing pulse oximetry?	_____________ minutes	

CRF 1 (neonate): Neonates admitted to SCBU

Hospital No: ______________________ Serial No: __ __ __ __ Nurse ID: __ __

State ID: __ Health Facility ID: __ __ Study ID: __ /__ __ /__ __ __ __ /__

PART B – General Admission Details			
**8**	Hospital Name		
**9**	Hospital Patient ID	__ __ __ __ __ __ __ __ __ __	
**10**	Date of Birth	__ __ / __ __ / __ __ __ __ (dd/mm/yyyy) Not recorded ☐99	
**11**	Gestation at birth<br>(provide as much detail as possible)	__ __ weeks, __ days Not recorded ☐99<br>☐1 Term ☐2 Preterm (<37 weeks) ☐3 Post term (>42 weeks)	
**12**	Birth weight	__ __ __ __ grams Not recorded ☐99	
**13**	Sex	☐1 Male ☐2 Female	
**14**	Date of Admission (dd/mm/yyyy)	__ __ / __ __ / __ __ __ __ (dd/mm/yyyy)	
**15**	Age at admission	__ __ days, __ __ hours Not recorded ☐99	
**16**	Admitted FROM	☐1 Born in this Hospital Not known ☐99<br>☐2 Transferred from other Health centre / Hospital<br>☐3 Brought from Home<br>☐4 Brought from Mission Home<br>☐3 Brought from Traditional Birth Attendant (TBA)	
**17**	Date of Discharge/death/abscond/transfer	__ __ / __ __ / __ __ __ __ (dd/mm/yyyy)	
**18**	Discharge weight	__ __ __ __ grams Not recorded ☐99	
**19**	Outcome	☐1 Discharged well<br>☐2 Died in hospital<br>☐3 Discharged unwell, recovery not expected<br>☐4 Discharged against medical advice (DAMA)<br>☐5 Absconded<br>☐6 Transferred to other hospital (specify):_____________	
**20**	Primary <u>admission</u> diagnosis<br>(main reason for admission recorded in the doctor's admission note)	1. __ __ None recorded ☐99<br>Other (specify): ______________	*See **Diagnosis Codes** on separate page*
**21**	Other <u>admission</u> diagnoses<br>(any additional diagnoses recorded in the doctor's admission note)	1. __ __ None recorded ☐99<br>2. __ __<br>3. __ __<br>4. __ __<br>Other (specify): ______________	
**22**	Primary <u>discharge</u> diagnosis<br>(main reason for admission recorded in the doctor's final case note)	1. __ __ None recorded ☐99<br>Other (specify): ______________	
**23**	Other <u>discharge</u> diagnoses<br>(any additional diagnoses recorded in the doctor's final case note)	1. __ __ None recorded ☐99<br>2. __ __<br>3. __ __<br>4. __ __<br>Other (specify): ______________	

PART C - Oxygen therapy		
24	Was SpO2 less than 90% (SpO2 <90%) at any time during admission?	☐1 YES ☐2 NO Not recorded ☐99
25	Was oxygen therapy given at any time during admission?	☐1 YES<br>☐2 NO => go to **PART D**
Starting oxygen		
26	Date that oxygen was <u>first started</u>*	__ __ / __ __ / __ __ __ __ (dd/mm/yyyy)
27	Last Sp02 recorded <u>before</u> oxygen was first started*	__ __ __% Not recorded ☐99
28	Flow rate when oxygen was first started*	__ L/min Not recorded ☐99
Stopping oxygen		
29	Date that oxygen was <u>finally ceased</u>*	__ __ / __ __ / __ __ __ __ (dd/mm/yyyy)<br>Oxygen was not ceased# ☐88<br>Not recorded ☐99
30	Last Sp02 recorded <u>before</u> oxygen was finally ceased*	__ __ __% Not recorded ☐99
31	First Sp02 recorded <u>after</u> oxygen was finally ceased*	__ __ __% Not recorded ☐99
32	What was the last Sp02 prior to <u>discharge/death/referral</u>?	__ __ __% Not recorded ☐99
Monitoring oxygen saturations (SpO2) while <u>on oxygen</u>		
33	While the child was on oxygen - were Sp02 readings recorded at least twice per day?	☐1 YES ☐2 NO
34	While the child was on oxygen - how many Sp02 readings **<85%**?	__ __
35	While the child was on oxygen - how many Sp02 readings **86-90%**?	__ __
36	While the child was on oxygen - how many Sp02 readings **91-95%**?	__ __
37	While the child was on oxygen - how many Sp02 readings **96-100%**?	__ __

* Oxygen may have been started and stopped multiple times during admission. "First started" means when the child when on oxygen for the first time. "Finally ceased" means when the child had oxygen stopped for the final time.
# "Oxygen was not ceased" means if the patient died, DAMA, absconded or was referred while still on oxygen.

CRF 1 (neonate): Neonates admitted to SCBU

Hospital No: ______________________ Serial No: __ __ __ __ Nurse ID: __ __

State ID: __ Health Facility ID: __ __ Study ID: __ /__ __ /__ __ __ __ /__

**PART D – Symptoms and Signs on Admission**				
**Signs and symptoms (at Admission)**				
**38**	Maternal fever during labour (>38 Celsius)	☐1 YES	☐2 NO	Not recorded ☐99
**39**	Prolonged rupture of membranes (>18 hours)	☐1 YES	☐2 NO	Not recorded ☐99
**40**	Offensive amniotic fluid (meconium stained)	☐1 YES	☐2 NO	Not recorded ☐99
**41**	Very poor feeding, or unable to suck	☐1 YES	☐2 NO	Not recorded ☐99
**42**	Lethargy, drowsy or unconscious (difficult to wake, moving only when stimulated)	☐1 YES	☐2 NO	Not recorded ☐99
**43**	Hypotonia (floppy), or spasms	☐1 YES	☐2 NO	Not recorded ☐99
**44**	High pitched crying, or inconsolable crying	☐1 YES	☐2 NO	Not recorded ☐99
**45**	Fast breathing (>60 breaths per minutes)	☐1 YES	☐2 NO	Not recorded ☐99
**46**	Severe respiratory distress<br>(e.g. grunting, gasping, severe chest indrawing)	☐1 YES	☐2 NO	Not recorded ☐99
**47**	Central cyanosis (blue lips or tongue)	☐1 YES	☐2 NO	Not recorded ☐99
**48**	Jaundice	☐1 YES	☐2 NO	Not recorded ☐99
**49**	Severe abdominal distension	☐1 YES	☐2 NO	Not recorded ☐99
**50**	Umbilical (or skin) redness or pus	☐1 YES	☐2 NO	Not recorded ☐99
**51**	Bulging fontanelle	☐1 YES	☐2 NO	Not recorded ☐99
**52**	Convulsions (seizures, fits)	☐1 YES	☐2 NO	Not recorded ☐99
**Measurements (at Admission)**				
**53**	Weight	__ __ __ __ grams		Not recorded ☐99
**54**	Height / length	__ __ cm		Not recorded ☐99
**55**	Head circumference (OFC)	__ __ cm		Not recorded ☐99
**56**	Heart rate (HR)	__ __ __ bpm		Not recorded ☐99
**57**	Respiratory rate (RR)	__ __ cpm		Not recorded ☐99
**58**	SpO2 on pulse oximetry (%)	__ __ __ %		Not recorded ☐99
**59**	Temperature (Celsius)	__ __ . __ Celsius		Not recorded ☐99
**60**	PCV (Packed Cell Volume)	__ __ %		Not recorded ☐99
**61**	Maternal HIV status	☐1 Positive	☐2 Negative	Not recorded ☐99

CRF 1 (neonate): Neonates admitted to SCBU

Hospital No: ____________________ Serial No: __ __ __ __ Nurse ID: __ __

State ID: __ Health Facility ID: __ __ Study ID: __/__ __/__ __ __ __/__

PART E – Neonatal Care		
**62**	Did the Admission Diagnosis indicate Preterm or Low Birth Weight (LBW)?	☐1 Preterm Not recorded ☐99<br>☐2 Very Preterm<br>☐3 Extremely Preterm<br>☐4 Low Birth Weight<br>☐5 Very Low Birth Weight
On the **FIRST DAY OF ADMISSION** (day 1), did the baby receive:		
**65**	Kangaroo mother care (skin to skin contact with mother)	☐1 YES ☐2 NO Not recorded ☐99
**66**	Temperature recorded at least three times per day (x3)	☐1 YES ☐2 NO Not recorded ☐99
**67**	Blood sugar recorded at least three times per day (x3)	☐1 YES ☐2 NO Not recorded ☐99
**68**	Respiratory rate (RR) and Heart rate (HR) recorded at least three times per day (x3)	☐1 YES ☐2 NO Not recorded ☐99
**69**	Apnoea monitoring? (using apnoea monitor, or continuous pulse oximeter)	☐1 YES ☐2 NO Not recorded ☐99
**70**	Feeding<br>(select all that apply)	☐1 Breastfeeding Not recorded ☐99<br>☐2 Cup and spoon (EBM)<br>☐3 Cup and spoon (formula)<br>☐4 Nasogastric feeds (EBM)<br>☐5 Nasogastric feeds (formula)<br>☐6 Intravenous (IV) fluids
**71**	Receive **antibiotics** (started on day 1)?	☐1 YES ☐2 NO / Not recorded **=> go to Q73**
**72**	➔ If yes, what dose, frequency, and duration?<br>(complete all that apply)<br>*See **Antibiotic codes** on separate page*<br><br>*Fill the corresponding **drug dose** in the table*	1 Drug: __ __<br>Frequency: __ times per day Not recorded ☐99<br>Duration: __ __ days Not recorded ☐99<br>2 Drug: __ __<br>Frequency: __ times per day Not recorded ☐99<br>Duration: __ __ days Not recorded ☐99<br>3 Drug: __ __<br>Frequency: __ times per day Not recorded ☐99<br>Duration: __ __ days Not recorded ☐99

	**DOSE**			
**DRUGS**	mg	ml	tab	mega unit
1				
2				
3				

At **ANY TIME** during admission, did the baby:		
**73**	Have apnoea? (prolonged stop breathing)	☐1 YES ☐2 NO Not recorded ☐99
**74**	Receive caffeine or aminophylline medication?	☐1 YES ☐2 NO / Not recorded **=> go to Q75**
**74b**	➔ If yes, what dose, frequency, and duration?<br>(complete all that apply)	Drug: __ __ Not recorded ☐99<br>Dose: __ __ ml Not recorded ☐99<br>Frequency: __ times per day Not recorded ☐99<br>Duration: __ __ days Not recorded ☐99
**75**	Have convulsions? (seizures, fits)	☐1 YES ☐2 NO Not recorded ☐99

Hospital No: ______________________ Serial No: __ __ __ __ Nurse ID: __ __

State ID: __ Health Facility ID: __ __ Study ID: __/__ __/__ __ __ __/__

76	Receive anticonvulsant medication? (e.g. phenobarbital, phenytoin, carbamazepine, valproate)	☐1 YES ☐2 NO / Not recorded **=> go to Q78**
77	➔ If yes, what dose, frequency, and duration? (complete all that apply)<br>*See **Anticonvulsant codes** on separate page*<br><br>*Fill the corresponding **drug dose** in the table*<br><table><tr><td></td><td colspan="2">**DOSE**</td></tr><tr><td>**DRUGS**</td><td>mg</td><td>ml</td></tr><tr><td>1</td><td></td><td></td></tr><tr><td>2</td><td></td><td></td></tr></table>	1 Drug: Drug: __ __<br>Frequency: __ times per day Not recorded ☐99<br>Duration: __ __ days Not recorded ☐99<br>2 Drug: Drug: __ __<br>Frequency: __ times per day Not recorded ☐99<br>Duration: __ __ days Not recorded ☐99
78	Receive Intravenous (IV) fluids?	☐1 YES ☐2 NO / Not recorded **=> go to Q80**
79	➔ If yes, what fluid and rate when first started, and for what duration?<br>*See **IV fluid codes** on separate page*	1 Fluid type: __ __ Not recorded ☐99<br>Rate: __ __ __ ml/hour Not recorded ☐99<br>Duration: __ __ days Not recorded ☐99<br><br>2 Fluid type: __ __ Not recorded ☐99<br>Rate: __ __ __ ml/hour Not recorded ☐99<br>Duration: __ __ day Not recorded ☐99<br><br>3 Fluid type: __ __ Not recorded ☐99<br>Rate: __ __ __ ml/hour Not recorded ☐99<br>Duration: __ __ day Not recorded ☐99
80	Receive Nasogastric (NG) fluids?	☐1 YES ☐2 NO / Not recorded **=> go to Q82**
81	➔ If yes, what fluid and rate when first started, and for what duration?<br>*See **NG fluid codes** on separate page*	1 Fluid type: __ __ Not recorded ☐99<br>Rate: __ __ __ ml/hour Not recorded ☐99<br>Duration: __ __ days Not recorded ☐99<br><br>2 Fluid type: __ __ Not recorded ☐99<br>Rate: __ __ __ ml/hour Not recorded ☐99<br>Duration: __ __ day Not recorded ☐99
82	Receive all three (3) Immunisations?<br>BCG, Oral Polio (OPV), Hepatitis B	☐1 YES ☐2 NO Not recorded ☐99
At the time of **DISCHARGE***, was the baby:		
83	Feeding well?	☐1 YES ☐2 NO Not recorded ☐99
84	Exclusively breastfed?	☐1 YES ☐2 NO Not recorded ☐99
85	Having high (>38 C) or low (<35.5) temperatures?	☐1 YES ☐2 NO Not recorded ☐99
86	Having any breathing problems?<br>(apnoea, difficult breathing, RR>60, chest indrawing)	☐1 YES ☐2 NO Not recorded ☐99

* If death/DAMA/abscond, leave questions 83-86 blank
