# Supplementary material for: Improving oxygen therapy for children and neonates in secondary hospitals in Nigeria: study protocol for a stepped-wedge cluster randomised trial
Source: Trials. 2017 Oct 27;18:502. doi: 10.1186/s13063-017-2241-8 (PMC5659007; doi:10.1186/s13063-017-2241-8)
Supplement: Supplementary file 2 — Data Collection Forms. (ZIP 2651 kb) [file 13063_2017_2241_MOESM2_ESM.zip › CRF2_Equipment_FINALR1.pdf]

CRF 2: Equipment

Hospital:______________ Visit Date:__ __ /__ __ /__ __ __ __

INSTRUCTIONS
**Specific instructions for CRF2 (Equipment)**
• *CRF2 (Equipment)* must be completed by Oxygen Project Engineers every three months at every hospital.<br>• Perform these checks in cooperation with the Nurse in charge and be respectful of patients and staff.
**General instructions**
• Answer every question. Use a black or blue ballpoint pen.<br>• Print all written entries with BLOCK CAPITAL LETTERS. Mark boxes with a cross (X) where requested (e.g. ☒).<br>• All date entries must appear in the format dd/mm/yyyy (e.g. 23/09/2016).<br>• If you make an error - draw a line through the error and write the correct value next to it. Date and initial the correction.

**PART A – General Details**			
**1**	Hospital Name		
**2**	Date of visit (dd/mm/yyyy)	__ __/__ __/__ __ __ __	
**3**	Name of Engineer		
**4**	How many Oxygen Project oxygen concentrators are there? Record all of their serial numbers.<br><br>Ensure that every concentrator is assessed and documented in Part B.	1.<br>2.<br>3.<br>4.<br>5.	
**5**	How many Oxygen Project pulse oximeters are there? Record all of their serial numbers.<br><br>Ensure that every oximeter is assessed and documented in Part C.	1.<br>2.<br>3.<br>4.<br>5.<br>6.<br>7.<br>8.<br>9.	
**6**	Which oxygen analyser was used? (serial number)<br>When was it last calibrated? (date)	Serial Number:	Date calibrated:<br>__ __/__ __/__ __ __ __
**General comments**			

CRF 2: Equipment

Hospital:______________ Visit Date:__ __ /__ __ /__ __ __ __

PART B - Concentrator Assessment							
1	**Concentrator serial number**	*E.g. 999*					
2	**Where is the concentrator located?**<br>Children's area (☐1 Child); Neonatal area (☐2 Neon); Combined Children & Neonatal area (☐3 Both)	☐1 Child<br>☒2 Neon<br>☐3 Both	☐1 Child<br>☐2 Neon<br>☐3 Both	☐1 Child<br>☐2 Neon<br>☐3 Both	☐1 Child<br>☐2 Neon<br>☐3 Both	☐1 Child<br>☐2 Neon<br>☐3 Both	☐1 Child<br>☐2 Neon<br>☐3 Both
3	**Has the weekly checklist been completed by users regularly over the past 3 months?**<br>☐1 Good = at least 9 weeks completed over the past 3 months<br>☐2 Mod = between 3 and 9 weeks completed over the past 3 months<br>☐3 Poor = less than 3 weeks completed over the past 3 months	☐1 Good<br>☒2 Mod.<br>☐3 Poor	☐1 Good<br>☐2 Mod.<br>☐3 Poor	☐1 Good<br>☐2 Mod.<br>☐3 Poor	☐1 Good<br>☐2 Mod.<br>☐3 Poor	☐1 Good<br>☐2 Mod.<br>☐3 Poor	☐1 Good<br>☐2 Mod.<br>☐3 Poor
4	**Is the concentrator clean and intact?**<br>☐1 Good = Clean and intact<br>☐2 Mod = Some dirt/dust, but no damage<br>☐3 Poor = Very dirty, or damage to external body	☐1 Good<br>☒2 Mod.<br>☐3 Poor	☐1 Good<br>☐2 Mod.<br>☐3 Poor	☐1 Good<br>☐2 Mod.<br>☐3 Poor	☐1 Good<br>☐2 Mod.<br>☐3 Poor	☐1 Good<br>☐2 Mod.<br>☐3 Poor	☐1 Good<br>☐2 Mod.<br>☐3 Poor
5	**How many hours are recorded on the meter?**	*164*					
6	**Does the 'Low Power Alarm' work?** It should be audible when the machine is first switched on. It should be audible if the machine is disconnected from the power while it is running.	☒1 YES<br>☐2 NO	☐1 YES<br>☐2 NO	☐1 YES<br>☐2 NO	☐1 YES<br>☐2 NO	☐1 YES<br>☐2 NO	☐1 YES<br>☐2 NO
7	**Is the 'Low Oxygen Concentration' alarm (yellow light) on when the concentrator is running normally?** It should be yellow when the machine is first switched on, and it should go off after a few minutes when it has reached >82% oxygen.	☒1 YES<br>☐2 NO	☐1 YES<br>☐2 NO	☐1 YES<br>☐2 NO	☐1 YES<br>☐2 NO	☐1 YES<br>☐2 NO	☐1 YES<br>☐2 NO
8	**Is the gas tubing and the connections intact and in place?** Check from the concentrator, to the Sureflow, to every bed. Use the 'bubble test' to check the flow at every individual flowmeter.	☐1 YES<br>☒2 NO	☐1 YES<br>☐2 NO	☐1 YES<br>☐2 NO	☐1 YES<br>☐2 NO	☐1 YES<br>☐2 NO	☐1 YES<br>☐2 NO
9	**What is the Oxygen Concentration at 2LPM flow?** If unable to get a reading, explain problem.	*93%*					
10	**What is the Oxygen Concentration at 5LPM flow?** If unable to get a reading, explain problem.	*90%*					

Concentrator Comments (describe problems identified, and what action will be taken) *[E.g. Cleaned concentrator. Retrained local staff and technician. Etc.]*

 Hospital:______________ Visit Date:__ __ /__ __ /__ __ __ __

PART C – Pulse Oximeter Assessment

1	**Oximeter serial number**	*E.g. 999*									
2	**Where is the oximeter located?**<br>Children's area (☐$_1$ Child); Neonatal area (☐$_2$ Neon);<br>Combined Children & Neonatal area (☐$_3$ Both)	☐1 Child<br>☒2 Neon<br>☐3 Both<br>☐4 Other	☐1 Child<br>☐2 Neon<br>☐3 Both<br>☐4 Other	☐1 Child<br>☐2 Neon<br>☐3 Both<br>☐4 Other	☐1 Child<br>☐2 Neon<br>☐3 Both<br>☐4 Other	☐1 Child<br>☐2 Neon<br>☐3 Both<br>☐4 Other	☐1 Child<br>☐2 Neon<br>☐3 Both<br>☐4 Other	☐1 Child<br>☐2 Neon<br>☐3 Both<br>☐4 Other	☐1 Child<br>☐2 Neon<br>☐3 Both<br>☐4 Other	☐1 Child<br>☐2 Neon<br>☐3 Both<br>☐4 Other	☐1 Child<br>☐2 Neon<br>☐3 Both<br>☐4 Other
3	**Is the oximeter clean and intact?**<br>☐$_1$ Good = Clean and intact<br>☐$_2$ Mod = Some dirt/dust, but no damage<br>☐$_3$ Poor = Very dirty, or damage to external body	☐1 Good<br>☒2 Mod.<br>☐3 Poor	☐1 Good<br>☐2 Mod.<br>☐3 Poor	☐1 Good<br>☐2 Mod.<br>☐3 Poor	☐1 Good<br>☐2 Mod.<br>☐3 Poor	☐1 Good<br>☐2 Mod.<br>☐3 Poor	☐1 Good<br>☐2 Mod.<br>☐3 Poor	☐1 Good<br>☐2 Mod.<br>☐3 Poor	☐1 Good<br>☐2 Mod.<br>☐3 Poor	☐1 Good<br>☐2 Mod.<br>☐3 Poor	☐1 Good<br>☐2 Mod.<br>☐3 Poor
4	**Is the oximeter charged and fully functional?** Turn on. Check the screen display. Test on your finger.	☒1 YES<br>☐2 NO	☐1 YES<br>☐2 NO	☐1 YES<br>☐2 NO	☐1 YES<br>☐2 NO	☐1 YES<br>☐2 NO	☐1 YES<br>☐2 NO	☐1 YES<br>☐2 NO	☐1 YES<br>☐2 NO	☐1 YES<br>☐2 NO	☐1 YES<br>☐2 NO
5	**SpO2 reading on your finger**	99%	__ __%	__ __%	__ __%	__ __%	__ __%	__ __%	__ __%	__ __%	__ __%

Oximeter Probes

6	**How many oximeter probes are available?** Test each probe.			
		**TOTAL**<br>__ __	**Functional**<br>__ __	**Not functional**<br>__ __
7	**Serial number of <u>non-functional</u> probes**<br>1.<br>2.<br>3.			
8	**Serial number of <u>replacement</u> probes**<br>1.<br>2.<br>3.			

Pulse Oximeter Comments (describe problems identified, and what action will be taken)
[E.g. Removed and replaced 'X' non-functional probes, and recorded their serial number. Returned to Lifebox for replacement under guarantee if life less than 1 year. Etc.]

PART D – Solar Assessment		
**Batteries and Inverter**		
**1**	Are the batteries stored in a secure location with adequate ventilation? (Describe location)	☐1 YES ☐2 NO ________________________________
**2**	How many batteries are in place?	__ __
**3**	Are the batteries and inverter clean and intact? (Clean if necessary. Check the top of batteries for corrosion, and apply grease if necessary)	☐1 Clean and intact<br>☐2 Some dirt/corrosion, but no damage<br>☐3 Very dirty/corroded or damage visible
**4**	What colour is the Charging Indicator? (If red, inform Dr Ajani the Solar Engineer)	☐1 Green solid (good, fully charged)<br>☐2 Green flashing (good, charging)<br>☐3 Yellow / Amber (not currently charging)<br>☐4 Red (problem)
**5**	INPUT from panels<br>- Voltage, $V_{i\ (panel)}$<br>- Amperage, $I_{i\ (panel)}$	__ __ Volts (V_i)<br>__ __ Amps (I_i)
**6**	State of Charge (SOC) of the batteries	__ __ %
**7**	OUTPUT from inverter<br>- Voltage, $V_{o\ (inverter)}$<br>- Amperage, $I_{o\ (inverter)}$	__ __ Volts (V_o)<br>__ __ Amps (I_o)
**8**	Time of recording (Use 24-hour clock, e.g. 1330)	Time: __ __ __ __ hours
**9**	Check the cables from <u>Batteries to Inverter</u>. (Should be cool/warm, but not hot. Look for signs of damage or disconnections)	☐1 Good - intact and not too hot<br>☐2 Problem (specify):
**10**	Check the cables from <u>Panels to Batteries</u>. (Should be cool/warm, but not hot. Look for signs of damage or disconnections)	☐1 Good - intact and not too hot<br>☐2 Problem (specify):
**Solar Panels**		
**11**	How many solar panels are in place?	__ __ __
**12**	Are the solar panels clean and intact?	☐1 Clean and intact<br>☐2 Some dirt/dust, but no damage<br>☐3 Very dirty, or damage visible
**Solar comments** (describe problems identified, and what action will be taken) *[E.g. Cleaned panels. Applied grease to batteries. Contacted Solar Provider. Etc.]*		
