# Supplementary material for: Improving oxygen therapy for children and neonates in secondary hospitals in Nigeria: study protocol for a stepped-wedge cluster randomised trial
Source: Trials. 2017 Oct 27;18:502. doi: 10.1186/s13063-017-2241-8 (PMC5659007; doi:10.1186/s13063-017-2241-8)
Supplement: Supplementary file 2 — Data Collection Forms. (ZIP 2651 kb) [file 13063_2017_2241_MOESM2_ESM.zip › Equipment Checklist_FINALR1.pdf]

Weekly Equipment Checklist

Hospital:___________________________

To keep the Oxygen Concentrator, Lifebox Pulse Oximeter, and other equipment functioning effectively please <u>perform these basic checks every week</u>, and <u>record on the Checklist</u>. If there are any problems, report them to the Engineer Responsible within 24 hours.

1. **Turn the Concentrator on. Does an audible alarm sound loudly for 5 seconds?** If the alarm is weak, or does not sound at all, contact the Engineer Responsible.
2. **Turn the Concentrator flowmeter to 5LPM.**
3. **Does the Oxygen Concentration indicator (yellow light) turn OFF after a few minutes?** If it stays on, the concentrator is not producing adequate oxygen and needs referral to the Engineer Responsible.
4. **Check the tubing connections of the Concentrator and Flowmeter assembly.** Tighten loose connections, and if there are cracks or leaks inform the Engineer Responsible.
5. **Submerge the nasal prongs in water and adjust the flow rate on the Flowmeter Assembly** (Sureflow). If bubbles are not produced appropriately at flow rates of 0.5-2.0 LPM, contact the Engineer Responsible. <u>Repeat for every flowmeter</u>. (You can now turn the concentrator off)
6. **Clean the concentrator body, check for damage, and record the hours of use.**
7. **Remove the external filter and replace with a clean, dry filter.** Wash the old filter and allow to dry.
8. **Check the Lifebox Pulse Oximeter on your finger, and check that each of the probes are working.** If any are not working, contact the Engineer Responsible.

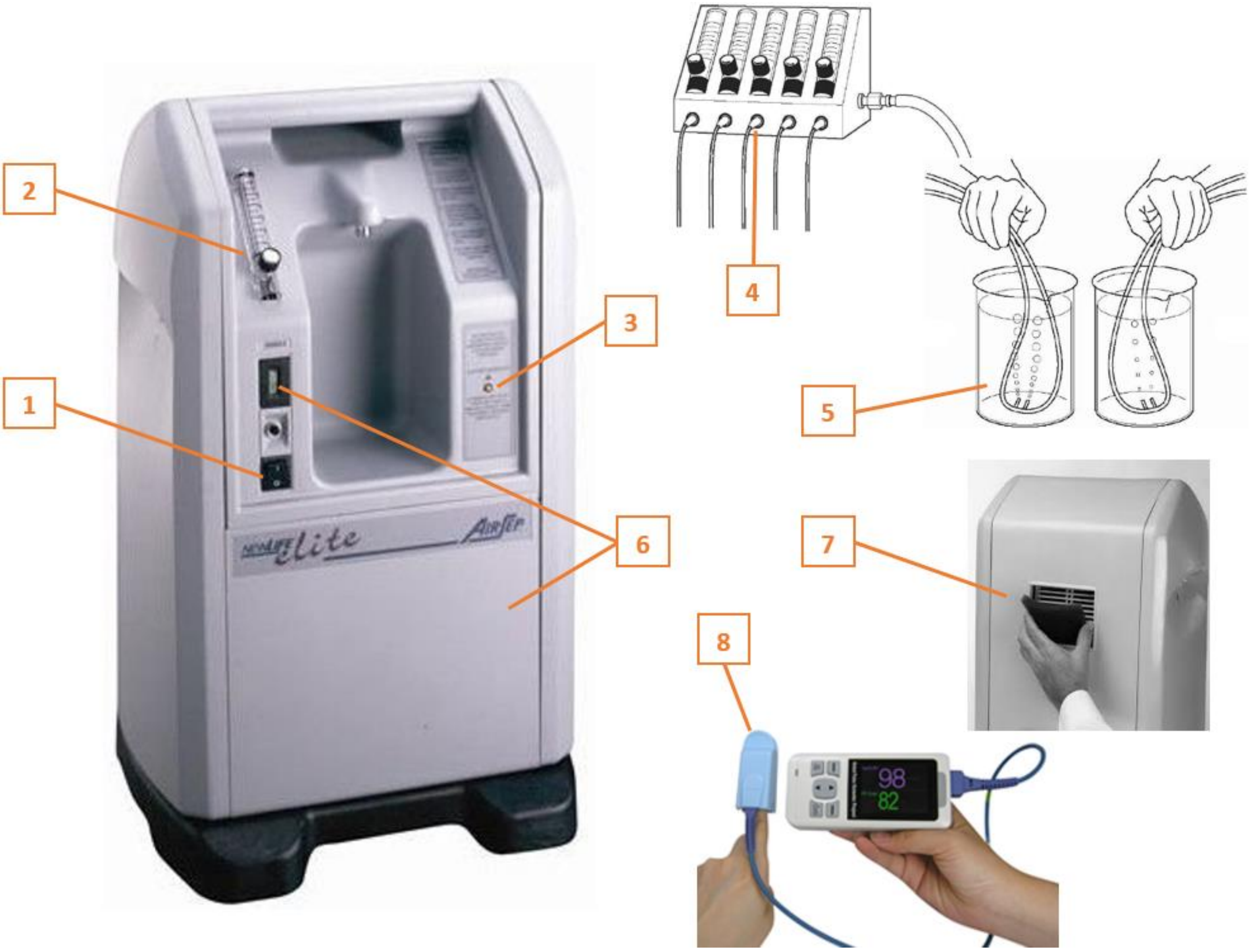

Weekly Equipment Checklist

Hospital:___________________________

Local Technician:
Contact:

Engineer Responsible:
Contact:

Equipment Serial Numbers

- Insert the serial numbers for all Oxygen Project equipment below and the date it was received.

Oxygen Concentrators

Date received	Concentrator Serial Number

Pulse Oximeters

Date received	Oximeter Serial Number

Oximeter Probes

Date received	Probe Serial Number

Weekly Equipment Checklist

Hospital:____________________________

April - June 2016

Complete this EVERY WEEK. Check every item of equipment. Indicate correct function of all equipment with ✓, and any problems with ✗.
Describe problems identified and action taken (including equipment serial number).

		Concentrators				Oximeters			
	**Checklist item ->**	①②③	④⑤	⑥	⑦	⑧		Document problems identified and action taken (include equipment serial number)	
**Week starting**	**Date**	**Alarms**	**Flow**	**Hours**	**Filter**	**Oximeter**	**Probes**	**Problems & Actions**	**Sign**
E.g.	*20/12/09*	✓	✓	4	✓	✓	✗	*Probe #1234567 broken, contacted Engineer for replacement.*	*HG*
4/4/16									
11/4/16									
18/4/16									
25/4/16									
2/5/16									
9/5/16									
16/5/16									
23/5/16									
30/5/16									
6/6/16									
13/6/15									
20/6/16									
27/6/16									

Weekly Equipment Checklist

Hospital:______________________

July - September 2016

Complete this EVERY WEEK. Check every item of equipment. Indicate correct function of all equipment with ✓, and any problems with ✗.
Describe problems identified and action taken (including equipment serial number).

		Concentrators				Oximeters		Document problems identified and action taken (include equipment serial number)	
	Checklist item ->	①②③	④⑤	⑥	⑦	⑧			
Week starting	Date	Alarms	Flow	Hours	Filter	Oximeter	Probes	Problems & Actions	Sign
E.g.	*20/12/09*	✓	✓	4	✓	✓	✗	*Probe #1234567 broken, contacted Engineer for replacement.*	*HG*
4/7/16									
11/7/16									
18/7/16									
25/7/16									
1/8/16									
8/8/16									
15/8/16									
22/8/16									
29/8/16									
5/9/16									
12/9/16									
19/9/16									
26/9/16									

Weekly Equipment Checklist

Hospital:____________________________

October - December 2016

Complete this <u>EVERY WEEK</u>. Check every item of equipment. Indicate correct function of all equipment with ✓, and any problems with ✗.
Describe problems identified and action taken (including equipment serial number).

		Concentrators				Oximeters			
	**Checklist item ->**	①②③	④⑤	⑥	⑦	⑧		Document problems identified and action taken (include equipment serial number)	
**Week starting**	**Date**	**Alarms**	**Flow**	**Hours**	**Filter**	**Oximeter**	**Probes**	**Problems & Actions**	**Sign**
E.g.	*20/12/09*	✓	✓	4	✓	✓	✗	*Probe #1234567 broken, contacted Engineer for replacement.*	*HG*
3/10/16									
10/10/16									
17/10/16									
24/10/16									
31/10/16									
7/11/16									
14/11/16									
21/11/16									
28/11/16									
5/12/16									
12/12/16									
19/12/16									
26/12/16									

Weekly Equipment Checklist

Hospital:____________________________

January – March 2017

Complete this EVERY WEEK. Check every item of equipment. Indicate correct function of all equipment with ✓, and any problems with ✗.
Describe problems identified and action taken (including equipment serial number).

		Concentrators				Oximeters			
	**Checklist item ->**	①②③	④⑤	⑥	⑦	⑧		Document problems identified and action taken (include equipment serial number)	
**Week starting**	**Date**	**Alarms**	**Flow**	**Hours**	**Filter**	**Oximeter**	**Probes**	**Problems & Actions**	**Sign**
E.g.	*20/12/09*	✓	✓	4	✓	✓	✗	*Probe #1234567 broken, contacted Engineer for replacement.*	*HG*
2/1/17									
9/1/17									
16/1/17									
23/1/17									
30/1/17									
6/2/17									
13/2/1									
20/2/17									
27/2/17									
6/3/17									
13/3/17									
20/3/17									
27/3/17									
